# Down-regulation of tomato *STEROL GLYCOSYLTRANSFERASE 1* perturbs plant development and facilitates viroid infection

**DOI:** 10.1093/jxb/erac361

**Published:** 2022-09-16

**Authors:** Adriana E Cisneros, Purificación Lisón, Laura Campos, Joan Manel López-Tubau, Teresa Altabella, Albert Ferrer, José-Antonio Daròs, Alberto Carbonell

**Affiliations:** Instituto de Biología Molecular y Celular de Plantas (Consejo Superior de Investigaciones Científicas–Universitat Politècnica de València), 46022 Valencia, Spain; Instituto de Biología Molecular y Celular de Plantas (Consejo Superior de Investigaciones Científicas–Universitat Politècnica de València), 46022 Valencia, Spain; Instituto de Biología Molecular y Celular de Plantas (Consejo Superior de Investigaciones Científicas–Universitat Politècnica de València), 46022 Valencia, Spain; Centre for Research in Agricultural Genomics (CSIC-IRTA-IAB-UB), Bellaterra, Barcelona, Spain; Centre for Research in Agricultural Genomics (CSIC-IRTA-IAB-UB), Bellaterra, Barcelona, Spain; Department of Biology, Healthcare and the Environment, Faculty of Pharmacy and Food Sciences, University of Barcelona, 08028 Barcelona, Spain; Centre for Research in Agricultural Genomics (CSIC-IRTA-IAB-UB), Bellaterra, Barcelona, Spain; Department of Biochemistry and Physiology, Faculty of Pharmacy and Food Sciences, University of Barcelona, 08028 Barcelona, Spain; Instituto de Biología Molecular y Celular de Plantas (Consejo Superior de Investigaciones Científicas–Universitat Politècnica de València), 46022 Valencia, Spain; Instituto de Biología Molecular y Celular de Plantas (Consejo Superior de Investigaciones Científicas–Universitat Politècnica de València), 46022 Valencia, Spain; University of Malaga, Spain

**Keywords:** Artificial microRNA, sterol glycosyltransferase, viroid, hypersusceptibility, *Solanum lycopersicum*

## Abstract

Potato spindle tuber viroid (PSTVd) is a plant pathogen naturally infecting economically important crops such as tomato (*Solanum lycopersicum*). Here, we aimed to engineer tomato plants highly resistant to PSTVd and developed several *S. lycopersicum* lines expressing an artificial microRNA (amiRNA) against PSTVd (amiR-PSTVd). Infectivity assays revealed that amiR-PSTVd-expressing lines were not resistant but instead hypersusceptible to the viroid. A combination of phenotypic, molecular, and metabolic analyses of amiRNA-expressing lines non-inoculated with the viroid revealed that amiR-PSTVd was accidentally silencing the tomato *STEROL GLYCOSYLTRANSFERASE 1* (*SlSGT1*) gene, which caused late developmental and reproductive defects such as leaf epinasty, dwarfism, or reduced fruit size. Importantly, two independent transgenic tomato lines each expressing a different amiRNA specifically designed to target *SlSGT1* were also hypersusceptible to PSTVd, thus demonstrating that down-regulation of *SlSGT1* was responsible for the viroid-hypersusceptibility phenotype. Our results highlight the role of sterol glycosyltransferases in proper plant development and indicate that the imbalance of sterol glycosylation levels favors viroid infection, most likely by facilitating viroid movement.

## Teaser

Depletion of glycosylated sterols in tomato plants induces developmental and reproductive defects such as leaf epinasty, dwarfism, and reduced fruit size, as well as hypersusceptibility to viroid infection.

## Introduction

Viroids are plant pathogens composed of a small (250–400 nucleotides), circular, single-stranded RNA genome without protein-coding capacity (Navarro *et al.*, 2021a). They induce symptoms in infected plants such as stunting, ­chlorosis, leaf curling, bark alterations, and size reduction of fruits and tubers (Flores *et al.*, 2005). Viroids replicate through an RNA-based rolling-circle mechanism (Branch and Robertson, 1984), move from cell to cell via plasmodesmata and over long distances through the phloem stream (Pallás and Gómez, 2017), and are classified into the *Pospiviroidae* or *Avsunviroidae* families, whose members replicate and accumulate in the nucleus and chloroplasts, respectively (Flores *et al.*, 2015; Daròs, 2016). Viroid pathogenesis is a complex process regulated by multiple factors including: (i) alteration of the expression of genes involved in the plant defense response, stress response, cell wall structure, and chloroplast function, among others (Owens *et al.*, 2017); (ii) the direct interaction of viroid genomic RNA with host factors (Adkar-Purushothama and Perreault, 2020); (iii) sequence-specific cleavage by viroid-derived small RNAs (vd-sRNAs) of complementary host mRNAs corresponding to genes involved in development; and (iv) the induction of ribosomal stress (Cottilli *et al.*, 2019). RNA-based resistance to viroids (reviewed in Dalakouras *et al.*, 2015; Flores *et al.*, 2017) has been achieved through the transgenic expression of antisense RNAs (Matoušek *et al.*, 1994), hammerhead ribozymes (Atkins *et al.*, 1995; Yang *et al.*, 1997; Carbonell *et al.*, 2011), dsRNA ribonucleases (Sano *et al.*, 1997), and hairpin RNAs of viroid sequence (Carbonell *et al.*, 2008; Schwind *et al.*, 2009; Adkar-Purushothama *et al.*, 2015), or by exogenous application of large amounts of dsRNA molecules of viroid sequence to leaves (Carbonell *et al.*, 2008). More recently, a large screening of multiple artificial microRNAs (amiRNAs) targeting sites distributed along viroid RNAs identified several amiRNAs that were highly active in agroinfiltrated leaves when co-expressed with an infectious viroid transcript (Carbonell and Daròs, 2017).

Plants contain three major species of sterols, named β-sitosterol, stigmasterol, and campesterol, and some members of the *Solanaceae* family also include important amounts of cholesterol (Moreau *et al.*, 2002; Benveniste, 2004). Sterols accumulate in different forms, as free sterols (FSs) with a free β-hydroxyl group at C-3 position on the backbone, conjugated esters, sterol glycosides (SGs), and acyl SGs. SGs are produced by UDP-glucose:sterol glycosyltransferases (SGTs), which catalyze the transfer of a glucose residue from UDP-glucose to the free hydroxyl group at position C-3 of FSs (Moreau *et al.*, 2002; Benveniste, 2004). Sterols are important for regulating plant growth and development, as changes in cellular sterol composition affect a variety of cellular processes, such as vascular and stomatal patterning, cell division, expansion, and polarity, cell-to-cell connectivity, and hormonal control, among others (Ramirez-Estrada *et al.*, 2017). Sterols are also key structural components of cellular membranes, regulate different membrane functions (e.g. passive or active transport across the membrane, activity of membranes associated with proteins), and changes in their relative proportion are known to alter membrane biophysical properties (Roche *et al.*, 2008; Grosjean *et al.*, 2015). Hence, sterols also play an important role in the plant response to a diverse list of abiotic and biotic stresses, including thermotolerance, drought, metal ions, hydrogen peroxide, and bacterial or fungal pathogens. However, the role of sterols in plant defense against viruses or viroids is unknown (Altabella *et al.*, 2022).

Here, we aimed to engineer tomato plants highly resistant to potato spindle tuber viroid (PSTVd), the type species of the *Pospiviroidae* family (Gross *et al.*, 1978). PSTVd naturally infects economically relevant crops such as potato (*Solanum tuberosum*) and tomato (*Solanum lycopersicum*), and is currently classified as a quarantine pathogen in certain regions of the world (Tsuda and Sano, 2014). A construct expressing a highly active anti-PSTVd amiRNA in *Nicotiana benthamiana* (Carbonell and Daròs, 2017) was introduced into tomato plants to generate multiple stably transformed lines that were analyzed for antiviroid resistance. Infectivity assays revealed, contrary to our expectations, that all amiRNA lines were hypersusceptible to PSTVd. A combination of phenotypic, molecular, and metabolic analyses of several amiRNA-expressing lines non-inoculated with the viroid revealed that the amiRNA was accidentally silencing the tomato *STEROL GLYCOSYLTRANSFERASE 1* (*SlSGT1*) gene, which caused late developmental and reproductive defects such as dwarfism, leaf epinasty, and reduced fruit size and weight. Moreover, two independent transgenic tomato lines, each expressing a different amiRNA specifically designed to target *SlSGT1*, were also hypersusceptible to PSTVd, thus confirming that down-regulation of *SlSGT1* was responsible for the hypersusceptibility phenotype.

## Materials and methods

### Plant materials and growing conditions

*Solanum lycopersicum* cv. Moneymaker Tm2 and cv. Micro-tom were grown in a growth chamber at 25 °C with a 12 light/12 h dark photoperiod. cv. Micro-tom transgenic lines expressing amiR-SlSGS1-1 or amiR-SlSGT1-2 amiRNAs were described elsewhere (Chávez *et al.*, 2023).

### DNA constructs

The *35S:amiR-PSTVd*, *35S:amiR-SlSGT1-1*, and *35S:amiR-SlSGT1-2* constructs were described previously (Carbonell and Daròs, 2017; Chávez *et al.*, 2023). The sequences of all amiRNA-generating precursors used in this study are listed in Supplementary Dataset S1.

### Generation and phenotyping of tomato cv. Moneymaker transgenic plants

*Agrobacterium tumefaciens* LBA4404 transformed with *35S:amiR-PSTVd* was co-cultured with tomato cotyledons. Explant preparation, selection, and regeneration were performed as previously described (Ellul *et al.*, 2003). Transformants were selected in hygromycin-containing medium, and then propagated in soil for seed production and for the infection studies. Non-transgenic controls (NTCs) were *in vitro*-regenerated tomato plants obtained in parallel with the transgenic plants. Phenotyping analyses were done using four NTCs and four independent amiRNA lines.

### Viroid infection assays

A CF11 cellulose-treated RNA extract was obtained from *N. benthamiana* tissue infected with PSTVd RG1 strain (GenBank accession no. U23058). Tomato plants were inoculated by mixing 5 μl of the infectious extract with 5 μl of a 10% carborundum solution (in 50 mM K_2_HPO_4_) on one leaf and evenly spreading the inoculum with a glass rod. After inoculation, plants were monitored for viroid symptom appearance for 4 weeks. The two youngest leaves were sampled for RNA preparation and analysis.

### RNA preparation

Total RNA from *S. lycopersicum* leaves was isolated in extraction buffer (1 M guanidinium thiocyanate, 1 M ammonium thiocyanate, 0.1 M sodium acetate, 5% glycerol, 38% water-saturated phenol), followed by chloroform extraction. RNA was precipitated in 0.5× isopropanol for 20 min. Triplicate samples from pools of two leaves were analyzed.

### Real-time RT–qPCR

Real-time reverse transcription followed by quantitative PCR (RT–qPCR) was done essentially as described by López-Dolz *et al.* (2020) in a QuantStudio 3 Real-Time PCR System (Thermo Fisher Scientific). Primers used for RT–qPCR are listed in Supplementary Table S1. Target RNA expression levels were calculated relative to two *S. lycopersicum* reference genes (*SlACT* and *SlEXP*) using the delta delta cycle threshold comparative method of the QuantStudio Design and Analysis Software (version 1.5.1.; Thermo Fisher Scientific).

### Small RNA blot assays

Total RNA (20 μg) was separated in 17% polyacrylamide gels containing 0.5× Tris/borate EDTA (TBE) and 7 M urea and transferred to a positively charged nylon membrane. Probe synthesis using [γ-^32^P]ATP (PerkinElmer) and T4 polynucleotide kinase (Thermo Fisher Scientific) and northern blot hybridizations were done at 38 °C in PerfectHyb Plus Hybridization Buffer (Sigma-Aldrich) as described previously (Montgomery *et al.*, 2008; Carbonell *et al.*, 2014). A Typhoon IP Imager System (Cytiva) was used to produce digital images from radioactive membranes. Oligonucleotides used as probes for sRNA blots are listed in Supplementary Table S1.

### 5ʹ-RLM-RACE

RNA ligase-mediated rapid amplification of 5ʹ cDNA ends (5ʹ-RLM-RACE) was done using the GeneRacer kit (Life Technologies) as described by Carbonell *et al.* (2015), except that the 5ʹ end of cDNA specific to *SlSGT1* was directly amplified in a single PCR using the GeneRacer 5ʹ and gene-specific AC-569 oligonucleotides. 5ʹ-RLM-RACE products were gel purified and cloned using the Zero Blunt TOPO PCR Cloning Kit (Life Technologies), introduced into *Escherichia coli* DH5α, screened for inserts, and sequenced. Control PCR reactions to amplify *SlACT* were done using oligonucleotides AC-280 and AC-281. The sequences of the oligonucleotides used are listed in Supplementary Table S1.

### Sterol analysis

For leaf sterol measurements, a pool of two apical leaves from four NTCs and four amiR-PSTVd independent *S. lycopersicum* lines was analyzed. Sterols were extracted and quantified by GC-MS in three technical replicates for each genotype (*n*=3), as previously described (Ramirez-Estrada *et al.*, 2017).

### RNA sequencing and data analysis

Total RNA from four wild-type and four amiR-PSTVd independent *S. lycopersicum* lines was analyzed for quantity, purity, and integrity with a 2100 Bioanalyzer (RNA 6000 Nano kit; Agilent), and submitted to BGI (Hong Kong, China) for strand-specific library preparation and mRNA sequencing (RNA-seq) in the DNBSEQ Platform. After quality analysis with FastQC (https://www.bioinformatics.babraham.ac.uk/projects/fastqc/), and adapter removal and low-quality trimming of raw reads with cutadapt (Martin, 2011), clean read pairs were mapped to the *S. lycopersicum* genome (version 4.0) using HISAT2 (Kim *et al.*, 2015), and non-uniquely mapped pairs were discarded. Numbers of read counts uniquely mapped to each tomato gene were obtained with htseq-count (Anders *et al.*, 2015) using the iTAG4.0 gene model annotation. Genes not having at least 1 TPM (transcripts per kilobase per million mapped reads) in either the four wild-type replicates or the four amiR-PSTVd lines were filtered out. Differential expression analysis was done with DESeq2 (Love *et al.*, 2014), with a false discovery rate of 1%. Differential gene expression analysis results are shown in Supplementary Dataset S2. An MA plot was generated using ggplot2 (Wickham 2016) in RStudio (https://www.rstudio.com/). Gene Ontology analysis was done using ShinyGo v0.75 (http://bioinformatics.sdstate.edu/go/) (Ge *et al.*, 2020) with a false discovery rate of 5%.

### Off-target analysis

TargetFinder v1.7 (https://github.com/carringtonlab/TargetFinder) (Fahlgren and Carrington, 2010) was used to obtain a ranked list of potential off-targets for amiR-PSTVd (Supplementary Dataset S3).

### Accession numbers

*Solanum lycopersicum* genes and corresponding locus identifiers are: *SlACT* (*Solyc04g011500.3*), *SlEXP* (SGN-U346908), *SlSGT1* (*Solyc06g007980.4*), *SlNAC082* (*Solyc11g005920.1*), and *SlPR1* (X71592.1).

## Results

### Transgenic tomato plants expressing an amiRNA against PSTVd are hypersusceptible to viroid infection

**Fig. 1.**
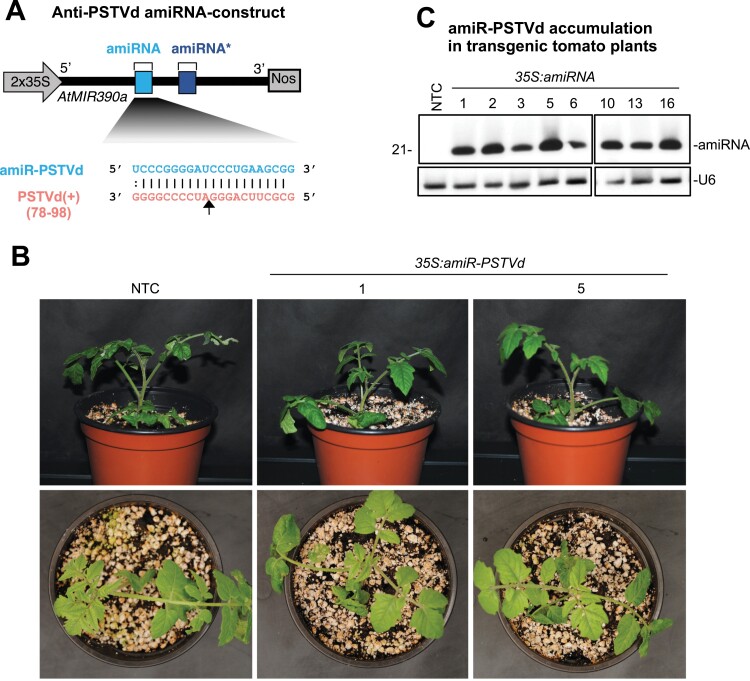
*Solanum lycopersicum* cv. Moneymaker T_1_ transgenic lines expressing amiR-PSTVd, an artificial microRNA (amiRNA) against potato spindle tuber viroid (PSTVd). (A) Diagram of the anti-PSTVd amiRNA construct, *35S:amiR-PSTVd*, engineered to express amiR-PSTVd from *Arabidopsis thaliana MIR390a* (*AtMIR390a*) precursor (in black). amiRNA and star strand positions in *AtMIR390a* are indicated in light blue and dark blue, respectively. Coordinates of the complete target site in PSTVd are given. The black arrow indicates the amiRNA-predicted cleavage site. (B) Photographs taken at 10 days post-transplanting of representative tomato lines expressing anti-PSTVd amiRNA and a non-transgenic control (NTC) plant. (C) Northern blot detection of amiR-PSTVd in RNA preparations from apical leaves of tomato plants. The U6 blot is shown as the loading control.

A construct expressing a highly effective anti-PSTVd amiRNA selected from a previous functional screening in *N. benthamiana* (Carbonell and Daròs, 2017) was introduced into *S. lycopersicum* cv. Moneymaker (Fig. 1A). Eight independent transgenic T_1_ lines were generated, all of which had a phenotype that was indistinguishable from that of NTC tomato plants at 10 days post-transplanting (Fig. 1B). Northern blot analysis of RNA preparations obtained from apical leaves revealed that all lines accumulated amiR-PSTVd to similar levels (Fig. 1C). Four of the lines (#1, #2, #5, and #16) were selected for further analyses.

**Fig. 2.**
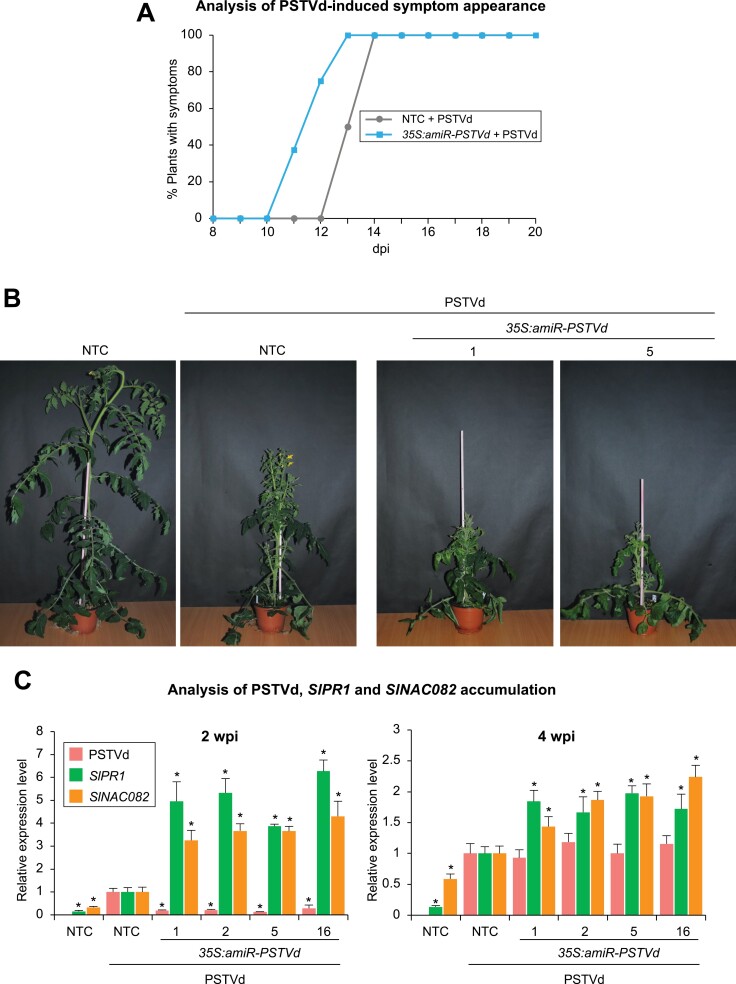
Functional analysis of the PSTVd amiRNA construct in transgenic *S. lycopersicum* plants. (A) Percentage of symptomatic amiR-PTSVd-expressing and NTC plants (*n*=3 per group) per day for 20 dpi. (B) Photographs taken at 4 wpi of representative transgenic tomato plants expressing amiR-PSTVd and NTC plants. (C) Accumulation of PSTVd, and of *SlPR1* and *SlNAC082* mRNAs, in tomato plants. Data are presented as the mean +SE relative levels of PSTVd (pink), and of *SlPR1* (green) and *SlNAC082* (orange) mRNAs, at 2 wpi (left panel) and at 4 wpi (right panel) after normalization to *ACTIN* (*SlACT*) and *EXPRESSED PROTEIN* (*SlEXP*), as determined by RT–qPCR (NTC+PSTVd=1 in all comparisons). For each target, asterisks indicate significant differences from NTC+PSTVd samples (*P<0.05; pairwise Student’s *t*-test comparisons).

To analyze the antiviroid resistance of each independent line, three individuals (propagated from cuttings) of each transgenic line were inoculated with PSTVd. In parallel, three NTC plants were mock- or PSTVd-inoculated. The appearance of typical PSTVd symptoms (leaf epinasty and chlorosis) in distant non-inoculated tissues was recorded during the 4 weeks post inoculation (wpi). Surprisingly, amiRNA plants displayed symptoms earlier than NTC plants (Fig. 2A). For instance, 37.5% of amiRNA plants showed symptoms as early as 11 days post inoculation (dpi), and 100% showed symptoms at 13 dpi. In contrast, in the NTC plants, the first symptoms were observed in 50% of the plants at 13 dpi, and only at 14 dpi were all the NTC plants symptomatic (Fig. 2A). At the end of the experiment (4 wpi), PSTVd-infected amiRNA lines were clearly shorter than NTC plants (Fig. 2B).

PSTVd accumulation was analyzed by RT–qPCR in selected amiRNA lines and NTCs at 2 wpi and 4 wpi (Fig. 2C). At 2 wpi, PSTVd accumulated to significantly lower levels in all amiRNA lines compared with infected NTCs, suggesting that amiR-PSTVd interferes with viroid infection, as predicted. However, this protective effect was transient because at 4 wpi PSTVd accumulation was similar in all infected plants (Fig. 2C). Importantly, the loss of the effect was not due to a decrease in amiR-PSTVd levels at later stages of infection, as amiR-PSTVd accumulated to similar levels at the two time points (Supplementary Fig. S1). Because viroid symptomatology in tomato positively correlates with the expression of pathogenesis-related (PR) proteins such as pathogenesis-related protein 1 (PR1) and ribosomal stress-inducing factors such as NAC082 (Cottilli *et al.*, 2019; Vázquez Prol *et al.*, 2021), the accumulation of *SlPR1* and *SlNAC082* mRNAs was analyzed in all plants (Fig. 2C). As expected, at 2 wpi both *SlPR1* and *SlNAC082* were induced in infected NTCs compared with mock NTCs. Interestingly, at the same time point, *SlPR1* and *SlNAC082* accumulated to significantly higher levels in all amiRNA lines. Similar results were obtained at 4 wpi, although the increase of *SlPR1* and *SlNAC082* levels in amiRNA lines relative to NTCs was more modest. All together, these results indicate that amiR-PSTVd-expressing lines are hypersusceptible to viroid infection. They also reveal that *SlPR1* and *SlNAC082* are induced in PSTVd-infected plants to levels that correlate with viroid symptomatology.

### AmiR-PSTVd-expressing lines display late developmental defects

**Fig. 3.**
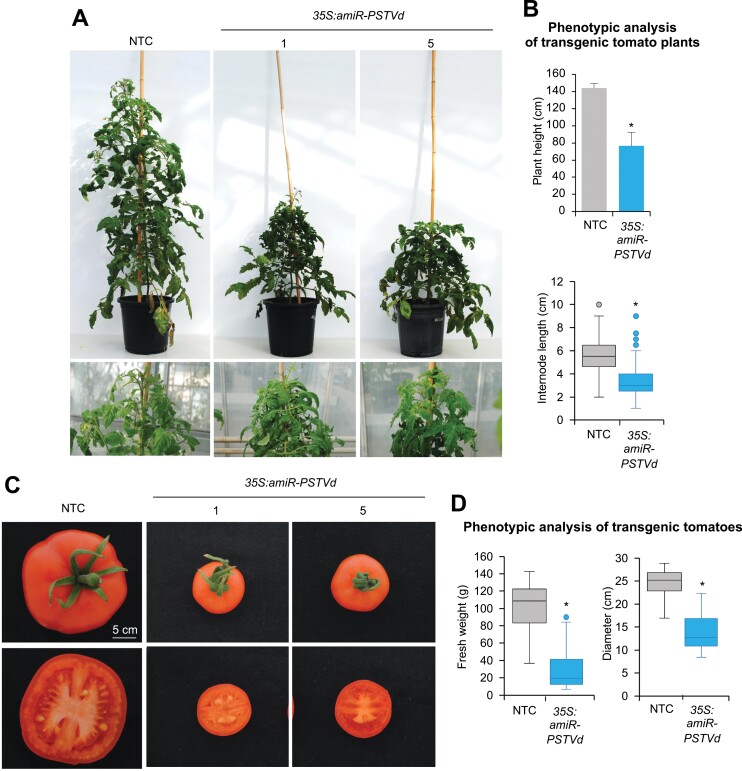
Phenotypic analysis of adult *S. lycopersicum* transgenic lines expressing an amiRNA against PSTVd. (A) Photographs taken at 8 wpt of two representative tomato T_1_ plants expressing anti-PSTVd amiRNA and an NTC plant. (B) Phenotypic analysis at 8 wpt of adult NTC plants and amiRNA transgenic lines. Data are presented as the mean +SD of plant height (upper panel) and the mean ±SD internode length (lower panel). (C) Photographs of representative tomato fruits from an NTC and two amiRNA lines. (D) Phenotypic analysis of tomato fruits from NTC and amiRNA lines. Data are presented as the mean ±SD of (left panel) fruit fresh weight and (right panel) fruit diameter. In B and D, asterisks indicate statistically significant differences from NTC samples (*P<0.05; pairwise Student’s *t*-test comparisons).

In parallel, NTCs and amiRNA lines were also grown for seed propagation in greenhouse conditions. Unexpectedly, at around 6 weeks post transplanting (wpt) of the *in vitro* ­plantlets, all amiRNA lines showed slight dwarfing and leaf curling, which became obvious at 8 wpt (Fig. 3A). The mean height of amiRNA plants was significantly reduced (1.9-fold) compared with the NTCs, and the internode length of the amiRNA lines was also significantly reduced compared with that of the NTCs (Fig. 3B). In addition, tomato fruits from the amiRNA lines were significantly smaller and had a lower fresh weight than fruits from NTCs (Fig. 3C, D). In conclusion, amiR-PSTVd expressing lines showed obvious developmental defects at later stages of growth, including dwarfism, leaf epinasty, and reduced fruit size and weight.

### Endogenous UDP-glucose:sterol glycosyl transferase 1 is down-regulated in amiR-PSTVd-expressing lines

The unexpected phenotype of the tomato transgenic lines prompted us to hypothesize that amiR-PSTVd might be accidentally targeting (and down-regulating) one or more ­endogenous transcripts involved in the induction of the observed late developmental defects. We reasoned that, if they exist, putative off-target(s) of amiR-PSTVd would have a high sequence complementarity with the amiRNA and be differentially underexpressed in amiR-PSTVd-expressing lines.

To reveal the identity of the putative off-target(s), first we used the TargetFinder tool (Fahlgren and Carrington, 2010) to generate a genome-wide list of all targets with a relatively high sequence complementarity with amiR-PSTVd (Supplementary Dataset S3). TargetFinder-predicted off-targets are ranked based on a Target Prediction Score (TPS) assigned to each amiRNA–target interaction. The TPS ranges from 1 to 11, that is, from the highest to the lowest degree of complementarity between the amiRNA and the putative target RNA. Indeed, amiR-PSTVd was designed with the P-SAMS amiRNA Designer tool (Fahlgren *et al.*, 2016) because none of the interactions between amiR-PSTVd and cellular RNAs had a TPS <4, which is the arbitrary cut-off that P-SAMS uses to assign high specificity to the designed amiRNA. However, we cannot completely rule out the possibility that amiR-PSTVd–target RNA interactions with a TPS >4 may be productive and lead to the down-regulation of the corresponding target RNA. Table 1 lists amiR-PSTVd most probable off-target transcripts with a TPS ≤6 based on TargetFinder analysis.

**Table 1.** Top putative amiR-PSTVd off-targets identified from target prediction analysis

Putative off-target	Description	TPS	Log_2_ fold change	*P*-adj	DE
*Solyc02g037540.3*	Disease resistance protein	4.5	–	–	–
*Solyc11g072610.3*	DNA repair (Rad51) family protein	5	–	–	–
*Solyc06g007980.4*	Sterol 3-beta-glycosyltransferase 1	5.5	–3.2536	3.32 × 10^–22^	Yes
*Solyc05g025900.3*	MT.1	5.5	–0.0092	0.9965	No
*Solyc11g005700.1*	RING-type E3 ubiquitin transferase	5.5	0.5618	0.1409	No
*Solyc02g084620.3*	Forkhead-associated (FHA) domain	6	–0.1747	0.7942	No
*Solyc05g053670.3*	60S ribosomal protein L13a	6	–0.1791	0.7614	No
*Solyc08g065940.5*	Zinc finger CCCH domain-containing protein 20	6	–	–	–
*Solyc10g078480.2*	Chitobiosyldiphosphodolichol beta-mannosyltransferase-like protein	6	–0.0367	0.9889	No
*Solyc12g009400.2*	Pyruvate dehydrogenase	6	–0.0517	0.9464	No
*Solyc12g009410.2*	Pyruvate dehydrogenase	6	–0.1918	0.5941	No
*Solyc01g103010.4*	Cullin-associated NEDD8-dissociated protein.1	6	–0.0384	0.9702	No

TPS, Target Prediction Score used by P-SAMS (Fahlgren *et al.* 2016); *P*-adj, adjusted *P*-value corrected for multiple comparisons; DE, differentially expressed, with a false discovery rate of 1%.

**Fig. 4.**
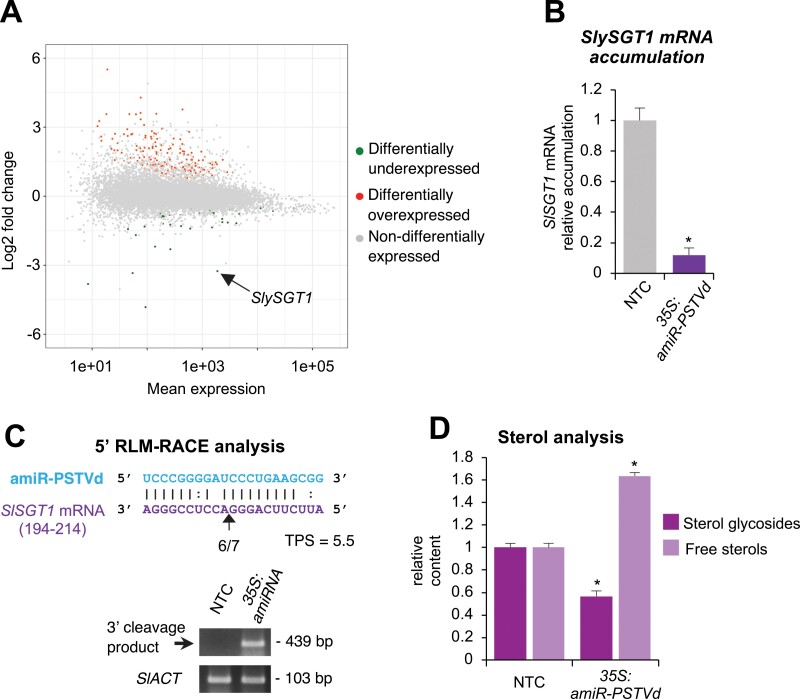
Molecular analysis of adult *S. lycopersicum* transgenic lines expressing an amiRNA against PSTVd. (A) MA plot showing log_2_ fold change versus mean expression of genes in four amiRNA lines (#1, #2, #5, and #16) compared with four NTC lines. Green, red, and grey dots represent differentially underexpressed, differentially overexpressed, and non-differentially expressed genes, respectively, in amiRNA lines compared with NTC plants. (B) Accumulation of *SlSGT1* mRNA in tomato plants. Data are presented as the mean +SE relative level of *SlSGT1* mRNAs at 8 wpt after normalization to *ACTIN* (*SlACT*) and *EXPRESSED PROTEIN* (*SlEXP*), as determined by RT–qPCR (NTC–1=1 in all comparisons). The asterisk indicates a significant difference from the NTC samples (*P<0.05; pairwise Student’s *t*-test comparison). (C) 5ʹ-RLM-RACE analysis of amiR-PSTVd-guided cleavage of *SlSGT1*. The upper panel shows the predicted base pairing between amiR-PSTVd and *SlSGT1* mRNA, and the expected amiR-PSTVd-based cleavage site is indicated by an arrow. The proportion of cloned 5ʹ-RLM-RACE products at the expected cleavage site is shown for amiR-PSTVd-expressing lines. TPS, Target Prediction Score. The lower panel shows an ethidium bromide-stained gel with 5ʹ-RLM-RACE products corresponding to the 3ʹ cleavage product from amiR-PSTVd-guided cleavage (top), and RT–PCR products corresponding to the control *SlACT* gene (bottom). The position and size of the expected amiRNA-based 5ʹ-RLM-RACE products are indicated, as well as the position and size of control RT–PCR products. (D) Sterol analysis. Data are presented as the mean +SD relative content of sterol glycosides and free sterols in NTC and amiRNA lines at 8 wpt. Asterisks indicate significant differences from NTC samples (*P<0.05; pairwise Student’s *t*-test comparisons).

Next, transcript libraries from four NTCs and four amiR-PSTVd-expressing lines were generated and analyzed. Differential gene expression analysis was done by comparing the transcript libraries from the NTCs with those from the amiR-PSTVd expressing lines. In total, 223 genes were differentially expressed in plants expressing amiR-PSTVd, with 176 genes being differentially overexpressed and 47 differentially underexpressed (Fig. 4A, Supplementary Dataset S2). Interestingly, *Solyc06g007980.4* was the only differentially underexpressed gene included in the list of most probable off-targets (Table 1). Indeed, this gene was the fifth most differentially underexpressed (Supplementary Dataset S2) and the third potential off-target with lowest TPS (Supplementary Dataset S3). This gene encodes the tomato sterol 3-beta glycosyltransferase 1 (SlSGT1), one of the four isoenzymes that catalyzes the glycosylation of the free hydroxyl group at the C-3 position of sterols to produce SGs in tomato (Ramirez-Estrada *et al.*, 2017). RT–qPCR analyses confirmed that *SlSGT1* mRNA accumulated to significantly lower levels in amiRNA-expressing lines compared with NTCs (Fig. 4B). Next, we used 5ʹ-RLM-RACE to test for amiR-PSTVd-directed off-target cleavage of *SlSGT1*. If amiR-PSTVd is cleaving *SlSGT1* mRNA, this analysis will detect the 3ʹ cleavage products. Cleavage products of the expected size (439 bp) were detected in samples expressing amiR-PSTVd, but not in NTC samples. Sequencing analysis confirmed that the majority (6 out of 7) of the sequences comprising these products contained the canonical 5ʹ end position predicted for amiR-PSTVd-guided cleavage (Fig. 4C).

Finally, SGs and FSs for the main tomato sterol species (cholesterol, campesterol, stigmasterol, and β-sitosterol) were analyzed and quantified in both NTC and amiRNA transgenic lines (Supplementary Table S2). As expected from *SlSGT1* down-regulation, the content of bulk SGs was significantly reduced by ~50% in amiRNA lines compared with NTCs (Fig. 4D). Conversely, the content of major FSs was significantly increased in amiRNA lines (Fig. 4D), thus confirming the reduced glycosylation activity in *SlSGT1*-silenced amiRNA lines. All together, these results indicate that amiR-PSTVd down-regulates *SlSGT1*, which affects sterol glycosylation and most likely causes the late developmental defects observed in the tomato transgenic lines.

### Is *SlSGT1* a direct target of PSTVd during infection?

**Fig. 5.**
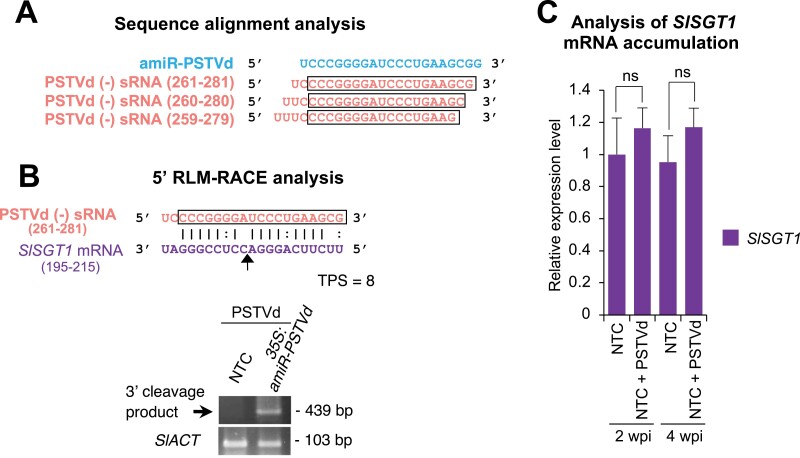
Analysis of putative down-regulation of *SlSGT1* mRNA in *S. lycopersicum* NTCs during PTSVd infection. (A) Sequence alignment between amiR-PSTVd and three PSTVd sRNAs of (–) polarity highly enriched in AGO1 immunoprecipitations. The sequence shared between each PSTVd sRNA and amiR-PSTVd is highlighted in a box. (B) 5ʹ RLM-RACE analysis of putative PSTVd sRNA-guided cleavage of *SlSGT1*. The upper panel shows the predicted base pairing between a PSTVd (–) sRNA and *SlSGT1* mRNA, and the expected cleavage site is indicated by an arrow. The lower panel shows an ethidium bromide-stained gel with 5ʹ-RLM-RACE products corresponding to the 3ʹ cleavage product from amiR-PSTVd-guided cleavage (top), and RT–PCR products corresponding to the control *SlACT* gene (bottom). (C) Mean +SE relative levels of *SlSGT1* mRNAs at 2 and 4 wpi after normalization to *ACTIN* (*SlACT*) and *EXPRESSED PROTEIN* (*SlEXP*), as determined by RT–qPCR (NTC 2 wpi=1 in all comparisons). ns, not statistically significant in the corresponding pairwise Student’s *t*-test comparison (*P>*0.05).

Notably, the late development defects of the amiRNA lines resembled the symptomatology observed in natural PSTVd infections. Thus, we hypothesized that *SlSGT1* might be targeted during PSTVd infection in tomato and that *SlSGT1* down-regulation may be, at least in part, responsible for the developmental defects observed in natural PSTVd infections. Because vd-sRNAs are known to associate with ARGONAUTE (AGO) proteins to target host genes (Navarro *et al.*, 2021a), we thought that maybe vd-sRNAs with a sequence similar to amiR-PSTVd may exist in natural PSTVd infections, associate with AGO1, and target *SlGST1*. Thus, we searched public high-throughput sRNA sequencing data and found three PSTVd-derived sRNAs of (–) polarity with high sequence similarity to amiR-PSTVd (Fig. 5A) that were in the top 10 of most highly enriched vd-sRNAs in AGO1-immunoprecipitated fractions from *N. benthamiana* tissue infected with PSTVd (Minoia *et al.*, 2014). Still, the lowest TPS from the interactions between PSTVd-sRNAs and *SlGST1* corresponded to PSTVd-sRNA (–) (261–281) with a relatively high score of 8. 5ʹ-RLM-RACE analysis confirmed that *SlSGT1* 3ʹ cleavage products could be detected only in infected transgenic lines expressing amiR-PSTVd, and not in infected NTCs (Fig. 5B). In addition, we observed that *SlSGT1* mRNA accumulation in NTC plants infected with PSTVd was not significantly different from that of mock-inoculated NTCs (Fig. 5C). Nonetheless, it is possible that *SlSGT1* mRNA accumulation remains unaltered because down-regulation by a putative PSTVd-sRNA may be compensated by an induction of *SlSGT1* transcription upon viroid infection. Thus, we also performed RT–qPCR with *SlSGT1* intron-specific primers and found that *SlSGT1* primary transcripts accumulate to similar levels in mock- and PSTVd-infected NTC plants (Supplementary Fig. S2). These results suggest that *SlSGT1* expression is not induced in PSTVd-infected plants. Taken together, our results do not support that *SlSGT1* may be a direct target in natural PSTVd infections in tomato.

### Transgenic tomato plants expressing an amiRNA specifically designed against *SlSGT1* are also hypersusceptible to viroid infection

**Fig. 6.**
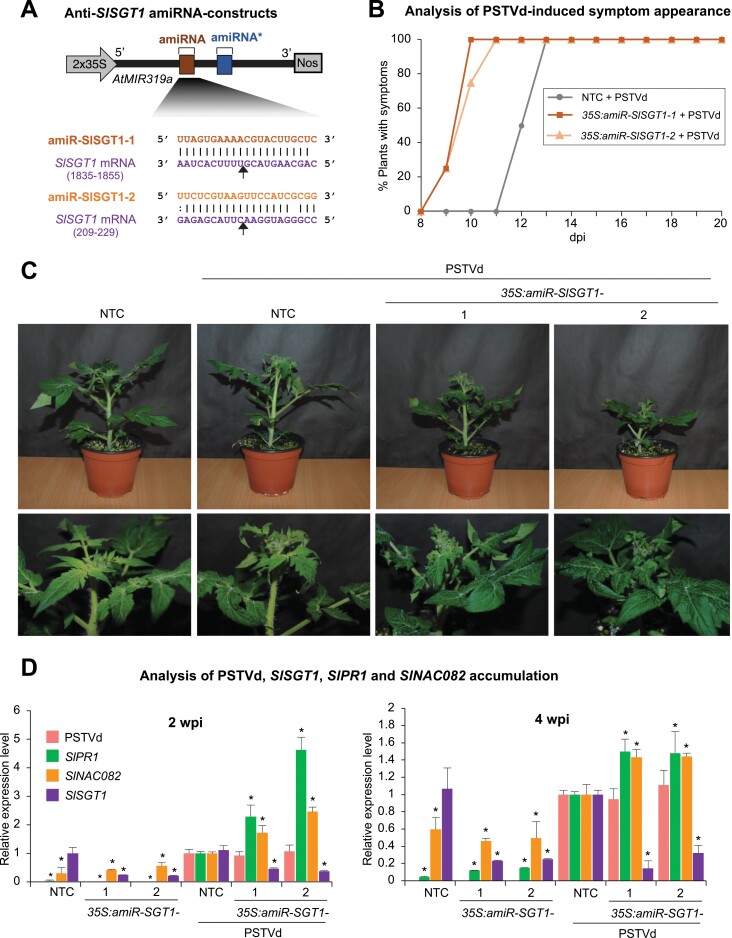
Functional analysis of *S. lycopersicum* cv. Micro-Tom transgenic lines expressing amiRNAs against *SlSGT1*. (A) Diagram of the two anti-SlSGT1 amiRNA constructs, *35S:amiR-SlSGT1-1* and *35S:amiR-SlSGT1-2*, engineered to express amiRNAs from *A. thaliana MIR319a* (*AtMIR319a*) precursor (in black). amiRNA and star strand positions in *AtMIR319a* are indicated in brown and dark blue, respectively. Coordinates of the complete target site in *SlSGT1* mRNA are given. The black arrows indicate the amiRNA-predicted cleavage sites. (B) Percentage of symptomatic amiR-SlSGT1-expressing and NTC plants (*n*=3 per group) per day for 20 dpi. (C) Photographs taken at 2 wpi of representative transgenic tomato plants expressing amiR-SlSGT1 and NTC plants. (D) Accumulation of PSTVd, and of *SlPR1*, *SlNAC082*, and *SlSGT1* mRNAs in tomato plants. Data are presented as the mean +SE relative levels of PSTVd (pink), and of *SlPR1* (green), *SlNAC082* (orange), and *SlSGT1* (purple) mRNAs, at 2 wpi (left panel) and at 4 wpi (right panel) after normalization to *ACTIN* (*SlACT*) and *EXPRESSED PROTEIN* (*SlEXP*), as determined by RT–qPCR (NTC+PSTVd=1 in all comparisons). For each target, asterisks indicate significant differences from NTC+PSTVd samples (*P<0.05; pairwise Student’s *t*-test comparisons).

To confirm that down-regulation of *SlSGT1* induces hypersusceptibility to PSTVd, two transgenic *S. lycopersicum* cv. Micro-Tom lines expressing amiR-SGT1-1 or amiR-SGT1-2 amiRNAs specifically designed to target *SlSGT1* (Fig. 6A) were challenged with PSTVd. Specifically, three individuals of each transgenic line and three NTCs were inoculated with PSTVd, and three other NTCs were mock inoculated. Plant response to PSTVd infection was analyzed as before, that is, recording the day of symptom appearance for each plant and analyzing the accumulation of PSTVd, and of *SlPR1* and *SlNAC082* mRNAs, at 2 wpi and 4 wpi. As a control, the accumulation of *SlSGT1* was also analyzed at the same time points.

As for the amiR-PSTVd lines, both amiR-SGT1-expressing lines displayed symptoms earlier than the NTC plants (Fig. 6B). For example, 75% of amiR-SGT1-1 and 100% of amiR-SGT1-2 plants showed symptoms as early as 10 dpi. In contrast, the first symptoms in NTC plants were observed in 50% of the plants at 12 dpi, and only at 13 dpi were all the NTC plants symptomatic (Fig. 6B). At the end of the experiment (4 wpi), both PSTVd-infected amiR-SGT1 lines were clearly more dwarfed than the NTC plants (Fig. 6C). RT–qPCR analysis showed that PSTVd accumulation was similar in all infected plants, at both 2 wpi and 4 wpi, whereas the accumulation of *SlPR1* and *SlNAC082* mRNAs was significantly greater in both amiR-SGT1 lines compared with the NTCs at the two time points (Fig. 6D). Finally, *SlSGT1* mRNA accumulation was significantly reduced in both amiR-SGT1 lines compared with the NTCs, in both the absence and the presence of PSTVd (Fig. 6D).

## Discussion

In this work, several *S. lycopersicum* cv. Moneymaker transgenic lines expressing an amiRNA against PSTVd (amiR-PSTVd) were generated. Phenotypic and molecular analyses revealed, contrary to our expectations, that amiR-PSTVd-expressing lines were not resistant but instead hypersusceptible to the viroid. Intriguingly, non-infected amiR-PSTVd lines showed late developmental defects including leaf epinasty, dwarfism, and reductions in fruit size and weight, which are also typical of PSTVd infection. A combination of transcriptome profiling with RT–qPCR, 5ʹ-RLM-RACE, and metabolomic analyses revealed that these phenotypic defects were caused by the accidental down-regulation of endogenous *SlGST1* by amiR-PSTVd-expressing lines.

### *SlSGT1* is required for proper plant and fruit development in tomato

Previous studies indicate that a proper balance between the levels of GSs and FSs is crucial for normal cell function and plant development. For instance, the ugt80A2,B1 Arabidopsis double mutant, which accumulates low GSs levels, displays diverse morphological and biochemical seed phenotypes (DeBolt *et al.*, 2009), alterations in the male gametophyte (Choi *et al.*, 2014), and aberrant root epidermal cell patterning (Pook *et al.*, 2017). In our work, down-regulation of *SlGST1* in amiR-PSTVd-expressing tomato plants induced late development phenotypes such as leaf epinasty, dwarfing, and reduced fruit size, similar to the phenotypes described in amiR-SGT1-expressing tomato plants (Chávez *et al.*, 2023). Shortened plant height and leaf area were previously observed in *Withania somnifera* plants transiently expressing a VIGS construct against endogenous *SGT*s (Singh *et al.*, 2016), whereas over-expression of *W. somnifera SGT1* caused enhanced growth and expansion of leaves (Saema *et al.*, 2016), thus reinforcing the idea of the essential role of SGTs in proper plant development. Here, as observed in our metabolic analyses, down-regulation of *SlSGT1* caused an imbalance of GSs and FSs which might alter membrane biophysical properties and negatively affect the function of membrane-localized proteins such as the receptors of the plant hormones cytokinins, ethylene, and brassinosteroids (Takeuchi *et al.*, 2021). Interference with hormone receptor function might affect the growth hormone signaling pathway, which could hinder the growth of amiR-PSTVd plants. Indeed, the dwarfing and leaf epinasty phenotype observed in the amiR-PSTVd lines is typical of plants affected in brassinosteroid signaling (Zhu *et al.*, 2013; Carbonell *et al.*, 2015). Yet, the significantly higher levels of free campesterol, the main precursor of brassinosteroids, in amiR-PSTVd lines (Supplementary Fig. S3) suggest that brassinosteroid biosynthesis may not be negatively affected at least by the altered campesterol levels.

On the other hand, a Gene Ontology analysis on our RNA-seq data revealed that several cellular component categories related to membranes were significantly altered (Supplementary Fig. S4; Supplementary Dataset S4), which is not surprising given the important roles of sterols in cellular membranes. Moreover, we identified several cytochrome P450-encoding genes that were misregulated (Supplementary Dataset S2). As cytochrome P450s are membrane-bound monooxygenases, the alteration of membranes due to *SlGST1* down-regulation may also directly affect the stability of these enzymes. Interestingly, several cytochrome P450 enzymes catalyze essential oxidative reactions in the biosynthesis or catabolism of several plant hormones, such as brassinosteroids, abscisic acid, jasmonyl-isoleucine, and strigolactones (Bak *et al.*, 2011). Pertinent to this context, our RNA-seq data indicate that CYP722 cytochrome P450 (*Solyc02g084930*), which is involved in brassinosteroid biosynthesis (Fujioka and Yokota, 2003; Nelson and Werck-Reichhart, 2011), is significantly down-regulated in amiR-PSTVd lines, while CYP707A1 (*Solyc08g005610*) and CYP94B3/CYP94C1 (*Solyc03g111290*/ *Solyc06g074420*), which participate in abscisic acid catabolism (Okamoto *et al.*, 2006) and jasmonyl-isoleucine inactivation and attenuation of the jasmonic acid signaling cascade (Bruckhoff *et al.*, 2016), respectively, are up-regulated in the same lines (Supplementary Dataset S2). Thus, it cannot be ruled out that altered cytochrome P450 balances may affect hormone biosynthesis or catabolic pathways. In any case, future research is needed to better dissect the effect of cellular membrane destabilization (due to reduced sterol glycosylation) on the stability of membrane components as well as the specific contribution of alterations in diverse hormone pathways in the phenotype of amiR-PSTVd lines.

### Viroid-induced symptoms positively correlate with *SlPR1* and *SlNAC082* expression levels but not with viroid titer

Interestingly, both amiR-PSTVd- and amiR-SlSGT1-expressing plants showing more severe symptoms than NTCs accumulated similar or lower levels of PSTVd at 2 wpi and 4 wpi (Figs 2C, 6D). These data indicate that symptom development is not correlated with PSTVd accumulation. Although this result might seem surprising, this observation was previously reported in experiments comparing the degree of symptoms induced by different PSTVd strains in tomato plants (Gruner *et al.*, 1995), and more recently also in *N. benthamiana*, *Prunus persica* (peach), and tomato plants infected with hop stunt viroid (Gomez *et al.*, 2008), peach latent mosaic viroid (Rodio *et al.*, 2006), or citrus exocortis viroid (Vázquez Prol *et al.*, 2020), respectively. In any case, our results suggest that viroid-induced symptoms in distant tissues depend on the presence of the viroid but do not correlate with viroid titer. This could indicate that symptom development in distant tissues may be triggered when a certain amount of viroid accumulates in this tissue above a threshold, but is not enhanced by further viroid accumulation.

Viroids trigger plant defense responses and induce the expression of PR proteins such as PR1 (Granell *et al.*, 1987; Lisón *et al.*, 2013), whose induction in tomato correlates with viroid-induced symptomatology, as has been observed for several viroids (Vázquez Prol *et al.*, 2021). Recently, the induction of the ribosomal stress mediator *SlNAC082* was observed upon CEVd infection in tomato and correlated with viroid-induced symptoms (Cottilli *et al.*, 2019; Vázquez Prol *et al.*, 2020), indicating that viroids can induce alterations in the biogenesis of ribosomes. Here, both *SlPR1* and *SlNAC082* were induced in PSTVd-infected NTCs and significantly over-induced in amiR-PSTVd- or amiR-SlGST1-expressing plants infected with PSTVd (Figs 2C, 6D).

### Viroid movement may be facilitated in *SlSGT1*-silenced plants

**Fig. 7.**
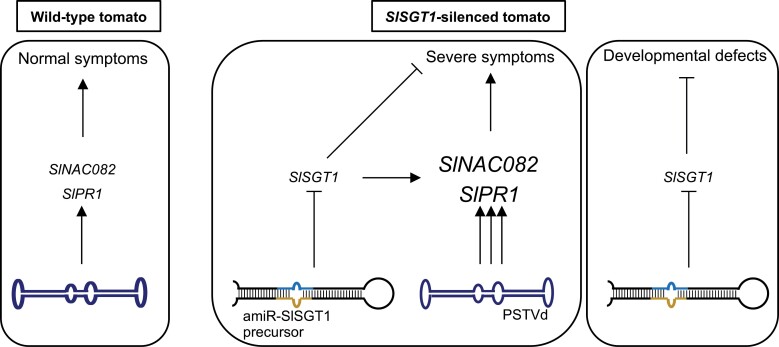
Model establishing the relationship between *SlSGT1*, *SlNAC082*, and *SlPR1* expression and PSTVd-induced symptomatology.

During infections, viroids move from inoculated to distal tissues, where symptoms such as leaf epinasty and chlorosis develop. Viroid movement includes intracellular movement (and subcellular compartmentalization for replication), exit of viroid progeny to neighboring cells, and entry to vascular tissue for long-distance trafficking (Pallás and Gómez, 2017). For instance, viroids can move from cell to cell via plasmodesmata, as was shown for PSTVd in tomato and tobacco mesophyll (Ding *et al.*, 1997). Our results show that *SlGST1* down-regulation impacts the cellular sterol profile (Supplementary Table S2), with a decrease in SGs and an increase in FSs (Fig. 4D). Because SGs and FSs are primary structural elements of cell membranes (Roche *et al.*, 2008; Grosjean *et al.*, 2015), it is possible that cytoplasmic membranes are altered in *SlSGT1*-silenced plants. This could affect membrane permeability and fluidity, and somehow facilitate cell-to-cell and long-distance transit of viroids through plasmodesmata, whose correct function depends on a specific membrane composition of lipids, including sterols (Grison *et al.*, 2015). Indeed, cytopathic effects at the plasma membrane in association with CEVd in infected *Gynura aurantiaca* were described more than four decades ago (Semancik and Vanderwoude, 1976). It is also of note that viroid systemic infections generally take several weeks and are slow compared with their virus counterparts infecting the same hosts, as viruses usually reach distal tissues in just a few days. This difference could be related to viroid steric difficulties in transiting the plasmodesmata in the absence of movement proteins, such as those encoded in plant virus genomes, that facilitate the process. It is tempting to speculate that the sterol imbalance may disturb plasmodesmal control, which could be used by viroids to move faster from cell to cell through plasmodesmata in infected tissues. In this scenario, PSTVd may reach distant tissues more rapidly in *SlSGT1*-silenced plants and so incite symptoms earlier than in wild-type plants. This could explain why symptom development was accelerated in *SlSGT1*-silenced plants (Fig. 7). However, our RT–qPCR and 5ʹ-RLM-RACE analyses in PSTVd-infected NTCs could not prove that *SlSGT1* is a direct target of PSTVd in natural infections. A close examination of recently published degradome datasets from PSTVd-infected tomato and *N. benthamiana* failed to identify degradome signatures of *SlSGT1* mRNAs by PSTVd-sRNAs (Navarro *et al.*, 2021b). Still, it is possible that both PSTVd-sRNAs and *SlSGT1* mRNAs may be accumulating in a non-uniform manner in tomato tissues such that cleaving of *SlSGT1* mRNAs might occur in only a subset of tissues. In this scenario, mRNA cleavage would be more difficult to detect than in the amiR-PSTVd plants, where amiRNA expression occurs in most (if not all) cells. In addition, *SlSGT1* silencing may need to occur in only certain cell types to induce PSTVd-like phenotypes. Finally, it is also possible that PSTVd-sRNAs could block *SlSGT1* translation or epigenetically regulate *SlSGT1* expression, or that other *SlSGT* isoforms could be regulated upon natural viroid infections. For all these reasons we cannot rule out the possibility that *SlSGT1* could be a direct target of PSTVd during natural infections.

### Roles of SGTs in the stress response

SGTs have been recently linked to enhanced salt stress resistance, as reported for Arabidopsis plants overexpressing the *W. somnifera SGT* gene (Mishra *et al.*, 2021). The role of SGTs in biotic stress responses has also been reported in several plant species. While silencing of several members of the *SGT* gene family led to a decrease in *W. somnifera* basal immunity against *Alternaria alternata* (Singh *et al.*, 2016), overexpression of *W. somnifera* SGT in Arabidopsis and tobacco plants was associated with increased resistance against *Alternaria brassicicola* and *Spodoptera litura*, respectively (Pandey *et al.*, 2014; Mishra *et al.*, 2017). It is not known whether the resistance effects in these reports were due to the modified levels of SGs or induced by the concomitant changes observed for other plant defense compounds. Pertinent to this observation, it was also described that resistance against the bacterial necrotroph *Botrytis cinerea* is enhanced in the Arabidopsis ugt80A2;B1 double mutant, which is impaired in SG biosynthesis. In this case the resistance phenotype was explained by the transcriptional and metabolic changes induced by an increase in jasmonic acid and camalexin levels observed in this double mutant (Castillo *et al.*, 2019).

Sterols have also been linked to plant virus replication (for a recent review see Altabella *et al.*, 2022), as the formation of viral replication complexes involves the recruitment of high levels of sterols. Moreover, it is known that the inhibition of sterol biosynthesis reduces tombusvirus replication in yeast and plants (Sharma *et al.*, 2010). In addition, it is possible that modifying the sterol profiles of a plant could ultimately increase its antiviral resistance, as suggested previously (Altabella *et al.*, 2022). Therefore, our amiR-PSTVd or amiR-SlSGT1 plants could be a very useful tool to assess in future experiments whether low *SlSGT1* levels induce enhanced antiviral resistance in tomato. A better understanding of the impact of sterol profiles on the biophysical properties of cellular membranes, and of the specific role of GSs predominant in Solanaceae species, will facilitate the development of enhanced antiviral resistance for next-generation crops.

## Supplementary data

The following supplementary data are available at *JXB* online.

Dataset S1. FASTA sequences of amiRNA-producing precursors.

Dataset S2. Differential expression analysis between NTC and *35S:amiR-PSTVd S. lycopersicum* leaf samples.

Dataset S3. amiR-PSTVd predicted off-targets.

Dataset S4. Gene Ontology datasets (differentially expressed genes in each biological process, cellular component, and molecular function category).

Fig. S1. Analysis of amiR-PSTVd accumulation during PSTVd infection in amiR-PSTVd-expressing tomato lines and NTCs.

Fig. S2. Accumulation of *SlSGT1* intron-containing primary transcripts in non-transgenic *S. lycopersicum* control plants (NTCs) during potato spindle tuber viroid infection.

Fig. S3. Campesterol analysis.

Fig. S4. Gene Ontology analysis.

Table S1. Name, sequence, and use of oligonucleotides used in this study.

Table S2. Content of glycosylated and free forms of cholesterol, campesterol, stigmasterol, and β-sitosterol in leaves of NTC plants and amiRNA lines.

erac361_suppl_Supplementary_Dataset_S1

erac361_suppl_Supplementary_Dataset_S2

erac361_suppl_Supplementary_Dataset_S3

erac361_suppl_Supplementary_Dataset_S4

erac361_suppl_Supplementary_Figures_S1-S4_Tables_S1-S2

## Data Availability

High-throughput sequencing data can be found in the Sequence Read Archive (SRA) database under accession number PRJNA849770. All other data supporting the findings of this study are available within the paper and within its supplementary data published online.

## References

[CIT0001] Adkar-Purushothama CR , Kasai A, Sugawara K, et al. 2015. RNAi mediated inhibition of viroid infection in transgenic plants expressing viroid-specific small RNAs derived from various functional domains. Scientific Reports 5, 17949. 26656294 10.1038/srep17949 PMC4677296

[CIT0002] Adkar-Purushothama CR , Perreault J-P. 2020. Current overview on viroid–host interactions. WIREs RNA 11, e1570. 31642206 10.1002/wrna.1570

[CIT0003] Altabella T , Ramirez-Estrada K, Ferrer A. 2022. Phytosterol metabolism in plant positive-strand RNA virus replication. Plant Cell Reports 41, 281–291. 34665312 10.1007/s00299-021-02799-x

[CIT0004] Anders S , Pyl PT, Huber W. 2015. HTSeq—a Python framework to work with high-throughput sequencing data. Bioinformatics 31, 166–169. 25260700 10.1093/bioinformatics/btu638 PMC4287950

[CIT0005] Atkins D , Young M, Uzzell S, Kelly L, Fillatti J, Gerlach WL. 1995. The expression of antisense and ribozyme genes targeting citrus exocortis viroid in transgenic plants. Journal of General Virology 76, 1781–1790. 9049383 10.1099/0022-1317-76-7-1781

[CIT0006] Bak S , Beisson F, Bishop G, Hamberger B, Höfer R, Paquette S, Werck-Reichhart D. 2011. Cytochromes P450. The Arabidopsis Book 2011, e0144. 10.1199/tab.0144 PMC3268508 22303269

[CIT0007] Benveniste P. 2004. Biosynthesis and accumulation of sterols. Annual Review of Plant Biology 55, 429–457. 10.1146/annurev.arplant.55.031903.141616 15377227

[CIT0008] Branch AD , Robertson HD. 1984. A replication cycle for viroids and other small infectious RNA’s. Science 223, 450–455. 6197756 10.1126/science.6197756

[CIT0009] Bruckhoff V , Haroth S, Feussner K, König S, Brodhun F, Feussner I. 2016. Functional characterization of CYP94-genes and identification of a novel jasmonate catabolite in flowers. PLoS One 11, e0159875. 27459369 10.1371/journal.pone.0159875 PMC4961372

[CIT0010] Carbonell A , Daròs J-A. 2017. Artificial microRNAs and synthetic *trans*-acting small interfering RNAs interfere with viroid infection. Molecular Plant Pathology 18, 746–753. 28026103 10.1111/mpp.12529 PMC6638287

[CIT0011] Carbonell A , Fahlgren N, Mitchell S, Cox KL Jr, Reilly KC, Mockler TC, Carrington JC. 2015. Highly specific gene silencing in a monocot species by artificial microRNAs derived from chimeric *miRNA* precursors. The Plant Journal 82, 1061–1075. 25809382 10.1111/tpj.12835 PMC4464980

[CIT0012] Carbonell A , Flores R, Gago S. 2011. *Trans*-cleaving hammerhead ribozymes with tertiary stabilizing motifs: *in vitro* and *in vivo* activity against a structured viroid RNA. Nucleic Acids Research 39, 2432–2444. 21097888 10.1093/nar/gkq1051 PMC3064770

[CIT0013] Carbonell A , Martinez de Alba AE, Flores R, Gago S. 2008. Double-stranded RNA interferes in a sequence-specific manner with the infection of representative members of the two viroid families. Virology 371, 44–53. 18028975 10.1016/j.virol.2007.09.031

[CIT0014] Carbonell A , Takeda A, Fahlgren N, Johnson SC, Cuperus JT, Carrington JC. 2014. New generation of artificial microRNA and synthetic trans-acting small interfering RNA vectors for efficient gene silencing in Arabidopsis. Plant Physiology 165, 15–29. 24647477 10.1104/pp.113.234989 PMC4012576

[CIT0015] Castillo N , Pastor V, Chávez Á, Arró M, Boronat A, Flors V, Ferrer A, Altabella T. 2019. Inactivation of UDP-glucose sterol glucosyltransferases enhances *Arabidopsis* resistance to *Botrytis cinerea*. Frontiers in Plant Science 10, 1162. 31611892 10.3389/fpls.2019.01162 PMC6776639

[CIT0016] Chávez A , Castillo N, López-Tubau JM, Atanasov KE, Fernández-Crespo E, Camañes G, Altabella T, Ferrer A. 2023. Tomato *STEROL GLYCOSYLTRANSFERASE 1* unveils a major role of steryl glycosydes in plant and fruit development. Environmental and Experimental Botany 206, 105181.

[CIT0017] Choi H , Ohyama K, Kim Y-Y, Jin J-Y, Lee SB, Yamaoka Y, Muranaka T, Suh MC, Fujioka S, Lee Y. 2014. The role of *Arabidopsis* ABCG9 and ABCG31 ATP binding cassette transporters in pollen fitness and the deposition of steryl glycosides on the pollen coat. The Plant Cell 26, 310–324. 24474628 10.1105/tpc.113.118935 PMC3963578

[CIT0018] Cottilli P , Belda-Palazón B, Adkar-Purushothama CR, Perreault J-P, Schleiff E, Rodrigo I, Ferrando A, Lisón P. 2019. Citrus exocortis viroid causes ribosomal stress in tomato plants. Nucleic Acids Research 47, 8649–8661. 31392997 10.1093/nar/gkz679 PMC6895259

[CIT0019] Dalakouras A , Dadami E, Wassenegger M. 2015. Engineering viroid resistance. Viruses 7, 634–646. 25674769 10.3390/v7020634 PMC4353907

[CIT0020] Daròs JA. 2016. Viroids: small noncoding infectious RNAs with the remarkable ability of autonomous replication. In: A Wang, X Zhou, eds. Current Research Topics in Plant Virology. Cham: Springer International Publishing, 295–321.

[CIT0021] DeBolt S , Scheible W-R, Schrick K, et al. 2009. Mutations in UDP-glucose:sterol glucosyltransferase in Arabidopsis cause transparent testa phenotype and suberization defect in seeds. Plant Physiology 151, 78–87. 19641030 10.1104/pp.109.140582 PMC2735980

[CIT0022] Ding B , Kwon M-O, Hammond R, Owens R. 1997. Cell-to-cell movement of potato spindle tuber viroid. The Plant Journal 12, 931–936. 9375403 10.1046/j.1365-313x.1997.12040931.x

[CIT0023] Ellul P , Garcia-Sogo B, Pineda B, Ríos G, Roig LA, Moreno V. 2003. The ploidy level of transgenic plants in *Agrobacterium*-mediated transformation of tomato cotyledons (*Lycopersicon esculentum* Mill.) is genotype and procedure dependent. Theoretical and Applied Genetics 106, 231–238. 12582848 10.1007/s00122-002-0928-y

[CIT0024] Fahlgren N , Carrington JC. 2010. miRNA target prediction in plants. Methods in Molecular Biology 592, 51–57. 19802588 10.1007/978-1-60327-005-2_4

[CIT0025] Fahlgren N , Hill ST, Carrington JC, Carbonell A. 2016. P-SAMS: a web site for plant artificial microRNA and synthetic trans-acting small interfering RNA design. Bioinformatics 32, 157–158. 26382195 10.1093/bioinformatics/btv534 PMC4681993

[CIT0026] Flores R , Hernández C, Martínez de Alba AE, Daròs JA, Di Serio F. 2005. Viroids and viroid-host interactions. Annual Review of Phytopathology 43, 117–139. 10.1146/annurev.phyto.43.040204.140243 16078879

[CIT0027] Flores R , Minoia S, Carbonell A, Gisel A, Delgado S, Lopez-Carrasco A, Navarro B, Di Serio F. 2015. Viroids, the simplest RNA replicons: how they manipulate their hosts for being propagated and how their hosts react for containing the infection. Virus Research 209, 136–145. 25738582 10.1016/j.virusres.2015.02.027

[CIT0028] Flores R , Navarro B, Kovalskaya N, Hammond RW, Di Serio F. 2017. Engineering resistance against viroids. Current Opinion in Virology 26, 1–7. 28738223 10.1016/j.coviro.2017.07.003

[CIT0029] Fujioka S , Yokota T. 2003. Biosynthesis and metabolism of brassinosteroids. Annual Review of Plant Biology 54, 137–164. 10.1146/annurev.arplant.54.031902.134921 14502988

[CIT0030] Ge SX , Jung D, Yao R. 2020. ShinyGO: a graphical gene-set enrichment tool for animals and plants. Bioinformatics 36, 2628–2629. 31882993 10.1093/bioinformatics/btz931 PMC7178415

[CIT0031] Gomez G , Martinez G, Pallas V. 2008. Viroid-induced symptoms in *Nicotiana benthamiana* plants are dependent on RDR6 activity. Plant Physiology 148, 414–423. 18599649 10.1104/pp.108.120808 PMC2528107

[CIT0032] Granell A , Bellés JM, Conejero V. 1987. Induction of pathogenesis-related proteins in tomato by citrus exocortis viroid, silver ion and ethephon. Physiological and Molecular Plant Pathology 31, 83–90.

[CIT0033] Grison MS , Brocard L, Fouillen L, et al. 2015. Specific membrane lipid composition is important for plasmodesmata function in Arabidopsis. The Plant Cell 27, 1228–1250. 25818623 10.1105/tpc.114.135731 PMC4558693

[CIT0034] Grosjean K , Mongrand S, Beney L, Simon-Plas F, Gerbeau-Pissot P. 2015. Differential effect of plant lipids on membrane organization: specificities of phytosphingolipids and phytosterols. Journal of Biological Chemistry 290, 5810–5825. 25575593 10.1074/jbc.M114.598805 PMC4342490

[CIT0035] Gross HJ , Domdey H, Lossow C, Jank P, Raba M, Alberty H, Sanger HL. 1978. Nucleotide sequence and secondary structure of potato spindle tuber viroid. Nature 273, 203–208. 643081 10.1038/273203a0

[CIT0036] Gruner R , Fels A, Qu F, Zimmat R, Steger G, Riesner D. 1995. Interdependence of pathogenicity and replicability with potato spindle tuber viroid. Virology 209, 60–69. 7747485 10.1006/viro.1995.1230

[CIT0037] Kim D , Langmead B, Salzberg SL. 2015. HISAT: a fast spliced aligner with low memory requirements. Nature Methods 12, 357–360. 25751142 10.1038/nmeth.3317 PMC4655817

[CIT0038] Lisón P , Tárraga S, López-Gresa P, Saurí A, Torres C, Campos L, Bellés JM, Conejero V, Rodrigo I. 2013. A noncoding plant pathogen provokes both transcriptional and posttranscriptional alterations in tomato. Proteomics 13, 833–844. 23303650 10.1002/pmic.201200286

[CIT0039] López-Dolz L , Spada M, Daròs J-A, Carbonell A. 2020. Fine-tune control of targeted RNAi efficacy by plant artificial small RNAs. Nucleic Acids Research 48, 6234–6250. 32396204 10.1093/nar/gkaa343 PMC7293048

[CIT0040] Love MI , Huber W, Anders S. 2014. Moderated estimation of fold change and dispersion for RNA-seq data with DESeq2. Genome Biology 15, 550. 25516281 10.1186/s13059-014-0550-8 PMC4302049

[CIT0041] Martin M. 2011. Cutadapt removes adapter sequences from high-throughput sequencing reads. EMBnet.journal 17, 10–12.

[CIT0042] Matoušek J , Schroder AR, Trnena L, et al. 1994. Inhibition of viroid infection by antisense RNA expression in transgenic plants. Biological Chemistry Hoppe-Seyler 375, 765–777. 7695839 10.1515/bchm3.1994.375.11.765

[CIT0043] Minoia S , Carbonell A, Di Serio F, Gisel A, Carrington JC, Navarro B, Flores R. 2014. Specific argonautes selectively bind small RNAs derived from potato spindle tuber viroid and attenuate viroid accumulation *in vivo*. Journal of Virology 88, 11933–11945. 25100851 10.1128/JVI.01404-14 PMC4178711

[CIT0044] Mishra MK , Srivastava M, Singh G, Tiwari S, Niranjan A, Kumari N, Misra P. 2017. Overexpression of *Withania somnifera SGTL1* gene resists the interaction of fungus *Alternaria brassicicola* in *Arabidopsis thaliana*. Physiological and Molecular Plant Pathology 97, 11–19.

[CIT0045] Mishra MK , Tiwari S, Misra P. 2021. Overexpression of *WssgtL3.1* gene from *Withania somnifera* confers salt stress tolerance in *Arabidopsis*. Plant Cell Reports 40, 2191–2204. 33523260 10.1007/s00299-021-02666-9

[CIT0046] Montgomery TA , Howell MD, Cuperus JT, Li D, Hansen JE, Alexander AL, Chapman EJ, Fahlgren N, Allen E, Carrington JC. 2008. Specificity of ARGONAUTE7-miR390 interaction and dual functionality in *TAS3 trans*-acting siRNA formation. Cell 133, 128–141. 18342362 10.1016/j.cell.2008.02.033

[CIT0047] Moreau RA , Whitaker BD, Hicks KB. 2002. Phytosterols, phytostanols, and their conjugates in foods: structural diversity, quantitative analysis, and health-promoting uses. Progress in Lipid Research 41, 457–500. 12169300 10.1016/s0163-7827(02)00006-1

[CIT0048] Navarro B , Flores R, Di Serio F. 2021a. Advances in viroid-host interactions. Annual Review of Virology 8, 305–325. 10.1146/annurev-virology-091919-092331 34255541

[CIT0049] Navarro B , Gisel A, Serra P, Chiumenti M, Di Serio F, Flores R. 2021b. Degradome analysis of tomato and *Nicotiana benthamiana* plants infected with potato spindle tuber viroid. International Journal of Molecular Sciences 22, 3725. 33918424 10.3390/ijms22073725 PMC8038209

[CIT0050] Nelson D , Werck-Reichhart D. 2011. A P450-centric view of plant evolution. The Plant Journal 66, 194–211. 21443632 10.1111/j.1365-313X.2011.04529.x

[CIT0051] Okamoto M , Kuwahara A, Seo M, Kushiro T, Asami T, Hirai N, Kamiya Y, Koshiba T, Nambara E. 2006. CYP707A1 and CYP707A2, which encode abscisic acid 8ʹ-hydroxylases, are indispensable for proper control of seed dormancy and germination in Arabidopsis. Plant Physiology 141, 97–107. 16543410 10.1104/pp.106.079475 PMC1459320

[CIT0052] Owens RA , Gómez G, Lisón P, Conejero V. 2017. Changes in the host proteome and transcriptome induced by viroid infection. In: Hadidi A, Flores R, Randles JW, Palukaitis P, eds. Viroids and Satellites. Boston: Academic Press, 105–114.

[CIT0053] Pallás V , Gómez G. 2017. Viroid movement. In: Hadidi A, Flores R, Randles JW, Palukaitis P, eds. Viroids and Satellites. Boston: Academic Press, 83–91.

[CIT0054] Pandey V , Niranjan A, Atri N, Chandrashekhar K, Mishra MK, Trivedi PK, Misra P. 2014. *WsSGTL1* gene from *Withania somnifera*, modulates glycosylation profile, antioxidant system and confers biotic and salt stress tolerance in transgenic tobacco. Planta 239, 1217–1231. 24610300 10.1007/s00425-014-2046-x

[CIT0055] Pook VG , Nair M, Ryu K, Arpin JC, Schiefelbein J, Schrick K, DeBolt S. 2017. Positioning of the SCRAMBLED receptor requires UDP-Glc:sterol glucosyltransferase 80B1 in *Arabidopsis* roots. Scientific Reports 7, 5714. 28720840 10.1038/s41598-017-05925-6 PMC5515990

[CIT0056] Ramirez-Estrada K , Castillo N, Lara JA, Arró M, Boronat A, Ferrer A, Altabella T. 2017. Tomato UDP-glucose sterol glycosyltransferases: a family of developmental and stress regulated genes that encode cytosolic and membrane-associated forms of the enzyme. Frontiers in Plant Science 8, 984. 28649260 10.3389/fpls.2017.00984 PMC5465953

[CIT0057] Roche Y , Gerbeau-Pissot P, Buhot B, Thomas D, Bonneau L, Gresti J, Mongrand S, Perrier-Cornet J-M, Simon-Plas F. 2008. Depletion of phytosterols from the plant plasma membrane provides evidence for disruption of lipid rafts. The FASEB Journal 22, 3980–3991. 18676403 10.1096/fj.08-111070

[CIT0058] Rodio ME , Delgado S, Flores R, Di Serio F. 2006. Variants of *Peach latent mosaic viroid* inducing peach calico: uneven distribution in infected plants and requirements of the insertion containing the pathogenicity determinant. Journal of General Virology 87, 231–240. 16361436 10.1099/vir.0.81356-0

[CIT0059] Saema S , Rahman L, Singh R, Niranjan A, Ahmad IZ, Misra P. 2016. Ectopic overexpression of *WsSGTL1*, a sterol glucosyltransferase gene in *Withania somnifera*, promotes growth, enhances glycowithanolide and provides tolerance to abiotic and biotic stresses. Plant Cell Reports 35, 195–211. 26518426 10.1007/s00299-015-1879-5

[CIT0060] Sano T , Nagayama A, Ogawa T, Ishida I, Okada Y. 1997. Transgenic potato expressing a double-stranded RNA-specific ribonuclease is resistant to potato spindle tuber viroid. Nature Biotechnology 15, 1290–1294. 10.1038/nbt1197-1290 9359114

[CIT0061] Schwind N , Zwiebel M, Itaya A, Ding B, Wang MB, Krczal G, Wassenegger M. 2009. RNAi-mediated resistance to *Potato spindle tuber viroid* in transgenic tomato expressing a viroid hairpin RNA construct. Molecular Plant Pathology 10, 459–469. 19523100 10.1111/j.1364-3703.2009.00546.x PMC6640329

[CIT0062] Semancik JS , Vanderwoude WJ. 1976. Exocortis viroid: cytopathic effects at the plasma membrane in association with pathogenic RNA. Virology 69, 719–726. 1258367 10.1016/0042-6822(76)90500-6

[CIT0063] Sharma M , Sasvari Z, Nagy PD. 2010. Inhibition of sterol biosynthesis reduces tombusvirus replication in yeast and plants. Journal of Virology 84, 2270–2281. 20015981 10.1128/JVI.02003-09 PMC2820916

[CIT0064] Singh G , Tiwari M, Singh SP, Singh S, Trivedi PK, Misra P. 2016. Silencing of sterol glycosyltransferases modulates the withanolide biosynthesis and leads to compromised basal immunity of *Withania somnifera*. Scientific Reports 6, 25562. 27146059 10.1038/srep25562 PMC4857139

[CIT0065] Takeuchi J , Fukui K, Seto Y, Takaoka Y, Okamoto M. 2021. Ligand–receptor interactions in plant hormone signaling. The Plant Journal 105, 290–306. 33278046 10.1111/tpj.15115

[CIT0066] Tsuda S , Sano T. 2014. Threats to Japanese agriculture from newly emerged plant viruses and viroids. Journal of General Plant Pathology 80, 2–14.

[CIT0067] Vázquez Prol F , López-Gresa MP, Rodrigo I, Bellés JM, Lisón P. 2020. Ethylene is involved in symptom development and ribosomal stress of tomato plants upon citrus exocortis viroid infection. Plants 9, 582. 32370199 10.3390/plants9050582 PMC7285140

[CIT0068] Vázquez Prol F , Márquez-Molins J, Rodrigo I, López-Gresa MP, Bellés JM, Gómez G, Pallás V, Lisón P. 2021. Symptom severity, infection progression and plant responses in Solanum plants caused by three pospiviroids vary with the inoculation procedure. International Journal of Molecular Sciences 22, 6189. 34201240 10.3390/ijms22126189 PMC8273692

[CIT0069] Wickham H. 2016. ggplot2: Elegant Graphics for Data Analysis. New York: Springer-Verlag.

[CIT0070] Yang X , Yie Y, Zhu F, Liu Y, Kang L, Wang X, Tien P. 1997. Ribozyme-mediated high resistance against potato spindle tuber viroid in transgenic potatoes. Proceedings of the National Academy of Sciences, USA 94, 4861–4865. 10.1073/pnas.94.10.4861 PMC24596 9144155

[CIT0071] Zhu JY , Sae-Seaw J, Wang ZY. 2013. Brassinosteroid signalling. Development 140, 1615–1620. 23533170 10.1242/dev.060590 PMC3621480

