# Supplementary material for: Down-regulation of tomato STEROL GLYCOSYLTRANSFERASE 1 perturbs plant development and facilitates viroid infection
Source: J Exp Bot. 2022 Sep 16;74(5):1564–78. doi: 10.1093/jxb/erac361 (PMC10010610; doi:10.1093/jxb/erac361)
Supplement: erac361_suppl_Supplementary_Dataset_S1 [file erac361_suppl_supplementary_dataset_s1.docx]

**Supplemental Dataset 1.** FASTA sequences of amiRNA-producing precursors. Sequences unique to the pri-miRNA, pre-miRNA, miRNA/amiRNA guide strand and miRNA*/amiRNA* strand sequences are highlighted in grey, white, blue and green, respectively.

**>*AtMIR390a*-based amiR-PSTVd precursor**

TATAGGGGGGAAAAAAAGGTAGTCATCAGATATATATTTTGGTAAGAAAATATAGAAATGAATAATTTCACGTTTAACGAAGAGGAGATGACGTGTGTTCCTTCGAACCCGAGTTTTGTTCGTCTATAAATAGCACCTTCTCTTCTCCTTCTTCCTCACTTCCATCTTTTTAGCTTCACTATCTCTCTATAATCGGTTTTATCTTTCTCTAAGTCACAACCCAAAAAAACAAAGTAGAGAAGAATCTGTATCCCGGGGATCCCTGAAGCGGATGATGATCACATTCGTTATCTATTTTTTCCGCTTCAGGTATCCCCGGGACATTGGCTCTTCTTACTACAATGAAAAAGGCCGAGGCAAAACGCCTAAAATCACTTGAGAATCAATTCTTTTTACTGTCCATTTAAGCTATCTTTTATAAACGTGTCTTATTTTCTATCTCTTTTGTTTAAACTAAGAAACTATAGTATTTTGTCTAAAACAAAACATGAAAGAACAGATTAGATCTCATCTTTAGTCTC

**>*AtMIR319a*-based amiR-SlSGT1-1 precursor**

ACAAACACACGCTCGGACGCATATTACACATGTTCATACACTTAATACTCGCTGTTTTGAATTGATGTTTTAGGAATATATATGTAGAGAACAAGTACGTTATCACTATTCACAGGTCGTGATATGATTCAATTAGCTTCCGACTCATTCATCCAAATACCGAGTCGCCAAAATTCAAACTAGACTCGTTAAATGAATGAATGATGCGGTAGACAAATTGGATCATTGATTCTCTTTGATTAGTGAAAACGTACTTGCTCTCTCTCTTTTGTATTCCAATTTTCTTGATTAATCTTTCCTGCACAAAAACATGCTTGATCCACTAAGTGACATATATGCTGCCTTCGTATATATAGTTCTGGTAAAATTAACATTTTGGGTTTATCTTTATTTAAGGCATCGCCATG

**>*AtMIR319a*-based amiR-SlSGT1-2 precursor**

ACAAACACACGCTCGGACGCATATTACACATGTTCATACACTTAATACTCGCTGTTTTGAATTGATGTTTTAGGAATATATATGTAGACCACGATGGAACTAACGAGATTCACAGGTCGTGATATGATTCAATTAGCTTCCGACTCATTCATCCAAATACCGAGTCGCCAAAATTCAAACTAGACTCGTTAAATGAATGAATGATGCGGTAGACAAATTGGATCATTGATTCTCTTTGATTCTCGTAAGTTCCATCGCGGTCTCTCTTTTGTATTCCAATTTTCTTGATTAATCTTTCCTGCACAAAAACATGCTTGATCCACTAAGTGACATATATGCTGCCTTCGTATATATAGTTCTGGTAAAATTAACATTTTGGGTTTATCTTTATTTAAGGCATCGCCATG
