# Supplementary material for: Down-regulation of tomato STEROL GLYCOSYLTRANSFERASE 1 perturbs plant development and facilitates viroid infection
Source: J Exp Bot. 2022 Sep 16;74(5):1564–78. doi: 10.1093/jxb/erac361 (PMC10010610; doi:10.1093/jxb/erac361)
Supplement: erac361_suppl_Supplementary_Figures_S1-S4_Tables_S1-S2 [file erac361_suppl_supplementary_figures_s1-s4_tables_s1-s2.pdf]

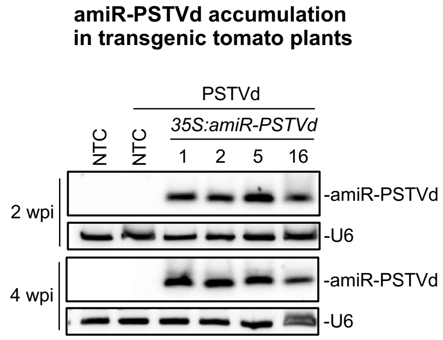

Supplemental Figure 1. Analysis of amiR-PSTVd accumulation during PSTVd infection in amiR-PSTVd-expressing tomato lines and non-transgenic controls (NTCs). Northern blot detection of amiR-PSTVd in RNA preparations from apical leaves of tomato plants at 2 and 4 weeks post-inoculation (wpi). The U6 blots are shown as loading controls.

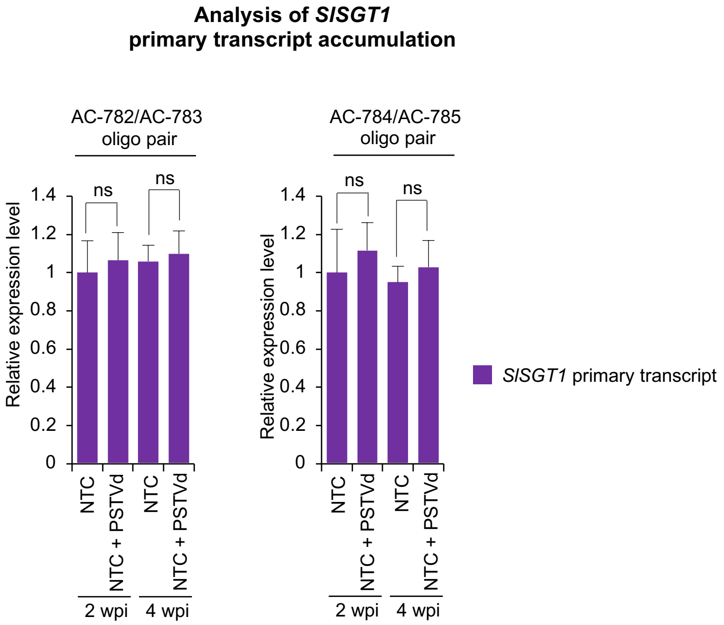

Supplemental Figure 2. Accumulation of *SlSGT1* intron-containing primary transcripts in non-transgenic *Solanum lycopersicum* control plants (NTCs) during *Potato spindle tuber viroid* infection. Bar graph shows mean relative level + standard error of *SlSGT1* primary RNAs at two and four weeks post inoculation (wpi) after normalization to *ACTIN* (*SlACT*), as determined by quantitative RT-PCR (RT-qPCR) (NTC 2 wpi = 1 in all comparisons) using two different oligo pairs (AC-782/AC-783 and AC-784/AC-785). ns refers to non-statistically significant in the corresponding pair-wise Students's t-test comparison. Other details are as in Figure 2C and 4C.

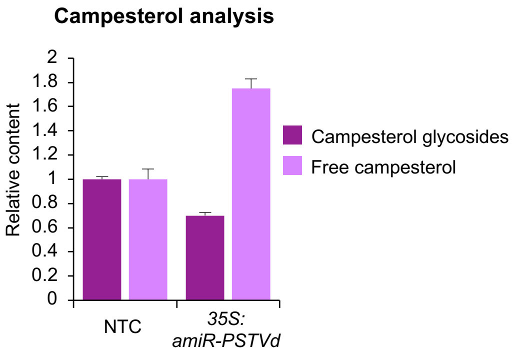

Supplemental Figure 3. Campesterol analysis. Bar graph show the mean relative content + standard deviation of campestrol glycosides (dark purple) and free campesterols (light purple) in NTC and amiRNA lines at 8 weeks post-transplanting.

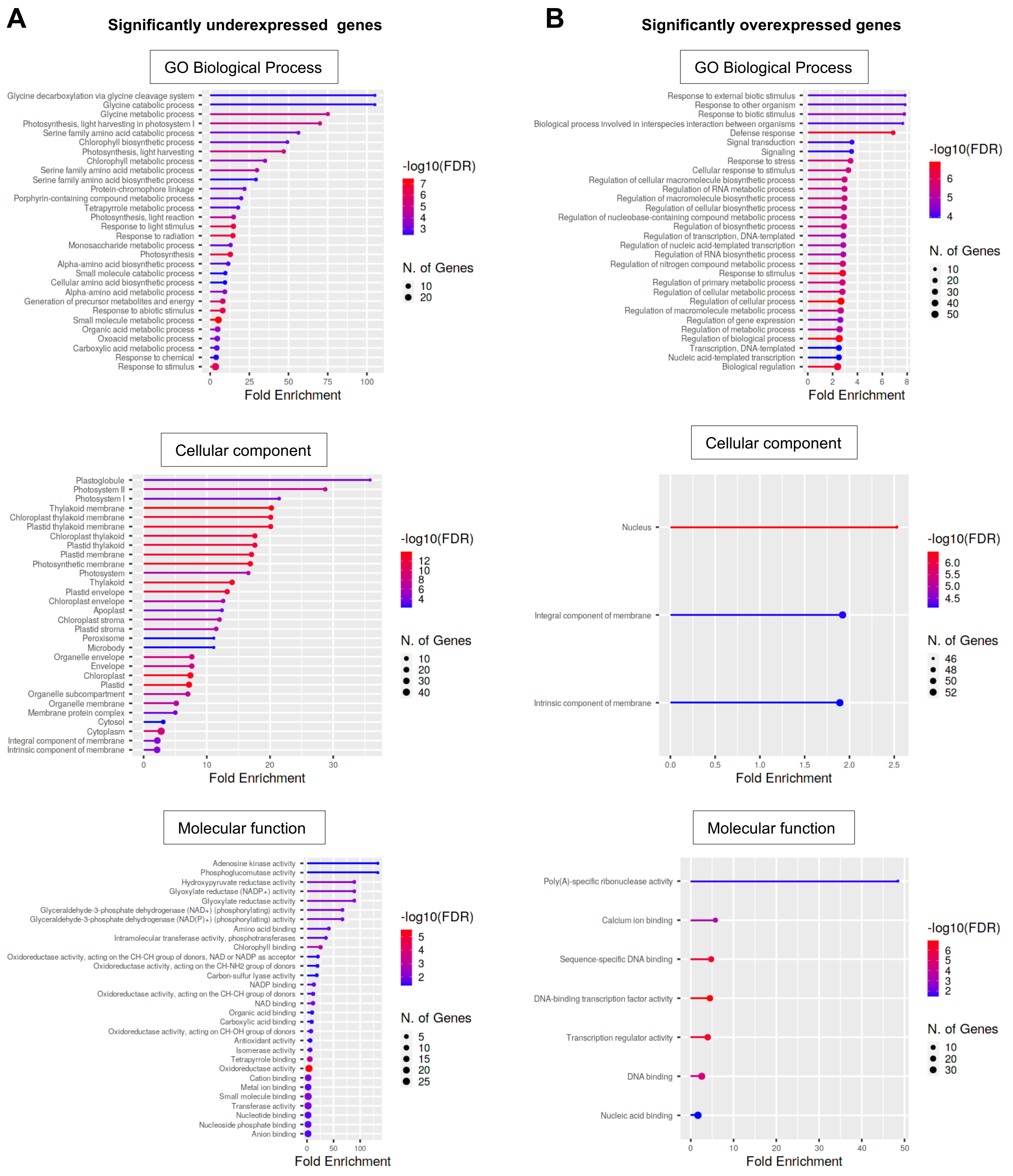

Supplemental Figure 4. Gene ontology analysis. Lollipot charts show the fold enrichment of differentially (FDR = 0.05) underexpressed (A) or overexpressed (B) genes in amiR-PSTVd lines compared to non-transgenic controls (NTCs) in each biological process, cellular component or molecular function category. the percentage of each molecular function category significantly altered in amiR-PSTVd lines compared to non-transgenic controls (NTCs).

Supplemental Table 1. Name, sequence and use of oligonucleotides used in this study.

**Oligonucleotide**	**Sequence**	**Construct/Aim**
AC-55	AGGGGCCATGCTAATCTTCTC	DNA probe for snoU6 detection
AC-280	CTAGGCTGGGTTCGCAGGAGATGATGC	*SlACT* qPCR
AC-281	GTCTTTTTGACCCATACCCACCATCACAC	
AC-408	GCCCCCTTTGCGCTGT	PSTVd-RG1 qPCR
AC-409	AAGCGGTTCTCGGGAGCTT	
AC-454	GCTAAGAACGCTGGACCTAATG	*SlEXP* qPCR
AC-455	TGGGTGTGCCTTTCTGAATG	
AC-456	C+CGC+TTC+AGG+GAT+CCC+CGG+GA	LNA probe for amiR-PSTVd detection
AC-569	CAGTAAAGGGCTGCACGTCACCAC	*SlGST1* PCR (5'RLM-RACE)
AC-575	AACGAGCCTCCACGATTGCCTAAA	*SlGST1* qPCR
AC-576	CTTTCTGGCATAAGAGCACCGCTA	
AC-581	ACTCAAGTAGTCTGGCGCAACTCA	*SlPR1* qPCR
AC-582	AGTAAGGACGTTGTCCGATCGAGT	
AC-583	TGCTGAAACCATTGGAACTG	*SlNAC082* qPCR
AC-584	CCAAGGAATTGCTTCCAAAA	
AC-782	GTGCCCAACTGTCTCTCTGA	*SlGST1* primary transcript qPCR
AC-783	ACACCACTGAACACTCGAAC	
AC-784	CGAAGTGCTTGGAACTGGAT	*SlGST1* primary transcript qPCR
AC-785	TACCTGTAGACGCTTGCCAA	
GeneRacer 5' Oligo	CGACTGGAGCACGAGGACACTGA	*SlGST1* PCR (5'RLM-RACE)
GeneRacer Oligo dT	GCTGTCAACGATACGCTACGTAACGGCATGACAG TGTTTTTTTTTTTTTTTTTTTTTTTT	cDNA synthesis (5'RLM-RACE)
GeneRacer RNA Oligo Adapter	CGACUGGAGCACGAGGACACUGACAUGGACUGAA GGAGUAGAAA	RNA ligation (5'RLM-RACE)

Supplemental Table 2. Content of glycosylated and free forms of cholesterol, campesterol, stigmasterol and β-sitosterol in leaves of NTC plants and amiRNA lines.

		Sterol glycosides (mg sterol/mg dry weight)			Free sterols (mg sterol/mg dry weight)		
		R1	R2	R3	R1	R2	R3
**NTC 1**	Cholesterol	0.0119	0.0116	0.0137	0.0155	0.0109	0.0142
	Campesterol	0.0030	0.0031	0.0023	0.0019	0.0015	0.0015
	Stigmasterol	0.0250	0.0207	0.0237	0.0174	0.0128	0.0156
	β-Sitosterol	0.0274	0.0222	0.0263	0.0126	0.0088	0.0102
**NTC 2**	Cholesterol	0.0153	0.0144	0.0127	0.0167	0.0130	0.0155
	Campesterol	0.0029	0.0032	0.0025	0.0019	0.0014	0.0013
	Stigmasterol	0.0305	0.0289	0.0229	0.0201	0.0183	0.0184
	β-Sitosterol	0.0331	0.0311	0.0262	0.0098	0.0083	0.0091
**NTC 3**	Cholesterol	0.0136	0.0102	0.0112	0.0274	0.0272	0.0289
	Campesterol	0.0027	0.0029	0.0033	0.0027	0.0029	0.0034
	Stigmasterol	0.0186	0.0141	0.0159	0.0495	0.0345	0.0378
	β-Sitosterol	0.0329	0.0365	0.0279	0.0250	0.0249	0.0258
**NTC 4**	Cholesterol	0.0167	0.0173	0.0171	0.0220	0.0320	0.0240
	Campesterol	0.0033	0.0036	0.0032	0.0013	0.0018	0.0022
	Stigmasterol	0.0283	0.0321	0.0279	0.0222	0.0305	0.0259
	β-Sitosterol	0.0404	0.0459	0.0370	0.0120	0.0156	0.0179
**amiR-PSTVd 1**	Cholesterol	0.0066	0.0081	0.0081	0.0171	0.0153	0.0155
	Campesterol	0.0019	0.0017	0.0021	0.0054	0.0035	0.0028
	Stigmasterol	0.0085	0.0106	0.0095	0.0517	0.0453	0.0442
	β-Sitosterol	0.0203	0.0150	0.0174	0.0304	0.0283	0.0263
**amiR-PSTVd 2**	Cholesterol	0.0083	0.0094	0.0117	0.0187	0.0174	0.0228
	Campesterol	0.0025	0.0016	0.0022	0.0038	0.0027	0.0030
	Stigmasterol	0.0086	0.0092	0.0118	0.0547	0.0476	0.0647
	β-Sitosterol	0.0211	0.0138	0.0169	0.0276	0.0220	0.0265
**amiR-PSTVd 5**	Cholesterol	0.0134	0.0137	0.0144	0.0218	0.0262	0.0193
	Campesterol	0.0032	0.0018	0.0026	0.0046	0.0042	0.0035
	Stigmasterol	0.0114	0.0107	0.0125	0.0664	0.0843	0.0630
	β-Sitosterol	0.0230	0.0206	0.0199	0.0385	0.0465	0.0285
**amiR-PSTVd 16**	Cholesterol	0.0077	0.0067	0.0099	0.0104	0.0115	0.0158
	Campesterol	0.0018	0.0018	0.0021	0.0028	0.0022	0.0032
	Stigmasterol	0.0098	0.0091	0.0109	0.0266	0.0337	0.0472
	β-Sitosterol	0.0217	0.0193	0.0206	0.0198	0.0262	0.0309
